# Testing the Effects of the Digital Linguistic Landscape on Engineering Education for Smart Construction

**DOI:** 10.1155/2022/4077516

**Published:** 2022-05-28

**Authors:** Lin Xu, Jingxiao Zhang, Yin Yuan, Junwei Zheng, Simon P. Philbin, Brian H. W. Guo, Ruoyu Jin

**Affiliations:** ^1^School of Foreign Languages, Northwest University, Xi'an 710127, China; ^2^School of Economics and Management, Chang'an University, Xi'an 710064, China; ^3^Faculty of Civil Engineering and Mechanics, Kunming University of Science and Technology, Kunming 650500, China; ^4^School of Engineering, London South Bank University, London SE10AA, UK; ^5^Department of Civil & Natural Resources Engineering, University of Canterbury, Christchurch, New Zealand; ^6^School of Built Environment and Architecture, South Bank University, London, UK

## Abstract

This study investigates the mechanism of digital linguistic landscapes in enabling engineering education for smart construction according to the educational dimensions of A (ability), S (skill), and K (knowledge). A questionnaire survey was conducted based on the core concepts of the informative dimension and symbolic dimension in digital language landscape as well as the ability dimension, knowledge dimension, and skill dimension in engineering education. Structural equation modeling (SEM) was used as the test method. The results of the research demonstrate that the informative dimension and symbolic dimension are two main aspects of DLL in education of engineering students for smart construction. Additionally, DLL has a significant positive impact on the ability, knowledge, and skill education of engineering students for smart construction. The research has theoretical and practical significance, as it not only enriches research on the relationship between DLL and engineering education for smart construction but also expands the theoretical understanding of engineering education from the perspective of linguistics. Furthermore, the study explores the path of the practical application of digital language landscape to engineering education for smart construction.

## 1. Introduction

The construction industry faces a number of pressing challenges, including improving the level of productivity and responding to the high level of fragmentation and complexity as well as leveraging the emerging opportunities by digital transformation [1, 2]. Indeed, the construction industry has already started to adopt digital technologies to improve the operational performance of industrial activities, including virtual reality, Internet of Things, and machine learning [3]. Moreover, the requirement for smart buildings is rapidly becoming an inherent constituent of policies associated with the design and development of buildings for the future [4]. Therefore, smart construction has been proposed as a new concept for the construction industry to adopt in order that the sector can fully capitalize on the opportunities afforded by digital transformation. Smart construction systems have huge potential to improve the efficiency of construction industry [5]. Specifically, they empower the engineering production system and promote the interconnection of engineering construction processes, including online and offline integration, resource, and element collaboration [6, 7]. In this context, the 21st century engineers and architects must be able to deal with the rapid pace of technological change and successfully navigate the highly interconnected world of industry [8]. Facing such profound changes in the construction industry triggered by digital technologies in the new era, there is an increasing demand for engineering students who are suitable for engaging in smart construction. Such students need to meet the requirements for the future development of the construction industry and to adapt to the transformation and upgrading of the industry [1, 3]. This also requires changes at the educational level [9].

The current basis for the education of engineering students suitable for smart construction fails to adequately integrate with digital technologies. There is a need to make changes to the engineering education system from different perspectives, including the digital linguistic landscape as well as aspects of the new industrial technology, information, and humanistic literacy. Therefore, it is necessary to change the traditional mode of educating smart construction engineering students to adopt a new approach that is suited to the digital context of the construction industry. Consequently, this study focuses on addressing the following question: Does the digital linguistic landscape play a role in the development of smart construction-related abilities, skills, and knowledge as part of engineering education? What is the mechanism of action?

Educating engineering students who possess smart construction capabilities faces various challenges, which can be summarized in four main areas. Firstly, the educational mode for enhancing the innovation awareness and ability of students is comparatively singular in nature. On this matter and in the case of China, Chen and Ding [10] identified that there is still a large gap between the innovation level of the country's construction industry and that of the developed countries, and this gap is especially prominent in the field of smart construction. Besides, university-level engineering students in general do not have a good understanding of the importance of innovation awareness and possess the capacity for innovation [11]. In addition, their awareness of the need to learn innovation skills and knowledge is currently limited [12]. In developing countries, educational programs have often been using out-of-date teaching methodologies, whereby students listen to lectures delivered by academic faculty without any practical applications or hands-on experience. This provokes a decrease in students' attention span and motivation and makes them become bored with the learning process [13, 14].

Secondly, the path for cultivating internationalized engineering students in the construction industry in China is somewhat narrow. In the context of the globalization trend and the “Belt and Road” initiative, there is a growing need for internationalized engineering students [15]. Engineering universities often prefer to establish long-term and stable cooperation with world-class universities, research institutions, and enterprises [16]. This kind of cooperation could provide good opportunities for engineering students in academic exchanges, engineering practice, and visits [17, 18]. However, resources are limited, and the number of students is large, while the opportunity to go abroad for exchange is not enough, which makes engineering students at this stage disconnected from the international advanced engineering concepts and academic ideas and often results in a lack of international vision and global awareness in engineering practice [19].

Thirdly, the knowledge horizon of engineering students in many cases is too narrow. China's engineering knowledge system needs improvement, and while the knowledge obtained by students is comprehensive, there are still difficulties in responding to industrial development trends and other arising technological challenges [20]. The current professional settings of engineering education are bounded by the traditional engineering disciplines, which often pay too much attention to the systemic nature of the disciplines themselves and ignore the interconnection between the knowledge across disciplines [21].

Fourthly, the digital linguistic landscapes are insufficient in number. The quantity of digital linguistic landscapes in the education environment of engineering students is far from enough. This not only serves to weaken the influence of the language environment itself on the cultivation of engineering students' consciousness, thinking, and cultural heritage [7] but also installs certain obstacles to the cognitive ability of such students [22]. This affects their learning ability and also decreases the significance of the digital information platform on the education of students suitable for smart construction through difficulties adapting to the rapid changes in the era of digitalization and information technology.

In view of this, digitalization is a way of information processing and representation and a technical method to store, transmit, process, handle, and apply information carriers (i.e., text, pictures, images, and signals) in digitally encoded form (usually binary). Digitalization is developing fast and has become a powerful tool for digital planning, construction, and operations, for instance, in the case of digital twins [23]. Digitalization and digital transformation have become a major research trend. Kohtamaki et al. [24] researched the relationship between digitalization and servitization, Ferreira et al. [25] studied the benefits of digitalization, and Verhoef et al. [26] researched the strategies of digital transformation. Digital linguistic landscape refers to the linguistic landscape in digital space. Linguistic landscapes have multiple roles in the development of students, as they can be powerful tools for education, language learning, critical thinking, language activism, and so on [27, 28]. Therefore, as an information platform for displaying the linguistic landscape through digital technology, digital linguistic landscape is a tool to broaden the influence of linguistic landscapes. The information provided by the digital linguistic landscape is rich, diversified, and internationalized, and its presentation media are diverse. Language and symbolic information on digital websites, digital signage, digital media, and other media all belong to digital linguistic landscapes. Therefore, the application of the digital linguistic landscape concept to the education of engineering students suitable for smart construction provides rich, diversified, and multilingual knowledge information and thereby strengthens the humanities of smart construction engineering students while also promoting the digital capabilities of engineering students.

The study aims to explore and test the mechanism of digital linguistic landscape on the education of engineering students suitable for smart construction through the use of the “ASK” (i.e., ability, skill, and knowledge) educational model based on the digital linguistic landscape platform. There is no relevant research on digital linguistic landscape for engineering talents cultivation at home and abroad, and this study has certain groundbreaking significance. This study has the following theoretical implications for the engineering education for smart construction: (1) defining the digital linguistic landscape; (2) expanding the theoretical scope of smart construction engineering education from the perspective of linguistics and providing the theoretical basis for the linguistic landscape atmosphere of the education; (3) exploring a new path to educate engineering students for smart construction and further enriching the research on the education path of smart construction-related engineering students.

## 2. Literature Review

### 2.1. Engineering Education for Smart Construction Students

According to the World Federation of Engineering Organization (WFOE), engineering capacity building, as a driver for sustainable socioeconomic development, has become a globally recognized priority [29]. Therefore, it is necessary to study the capacities of smart construction engineering students.

A search of the keywords “smart construction engineering students' qualities” and “abilities” in domestic and foreign mainstream databases showed that there was no relevant literature. In order to summarize the core qualities of engineering students suitable for smart construction more accurately, the core competence standards for graduates of the Washington Accord, Sydney Accord, and FEANI, as well as the domestic standards for the connotation of engineering students' qualities, were analyzed and extracted. This was carried out from the perspective of professional certification of engineering education at home and abroad, focusing on the keywords “smart construction,” “engineering students,” “qualities,” “competence,” and “abilities.” After carefully screening the relevant literature, 10 of the core qualities were finally selected to analyze and summarize the core qualities of engineering students, as shown in Table 1.

From Table 1, it can be observed that, in the context of “New Engineering,” the quality of engineering students mainly focuses on lifelong learning ability, engineering innovation ability, engineering thinking ability, interdisciplinary and integration ability, communication, and cooperation ability, as well as engineering knowledge and professional skills. On this basis and combined with the new requirements for professional students relevant to smart construction [1, 40–44], the core qualities of engineering students' in smart construction were summarized as follows:

- Engineering innovation ability: innovation is critical to economic and social prosperity [45]. Educating engineering students for smart construction who are innovative is the key to building a dynamic and successful economy [12]. The focus of cultivating the innovation ability of engineering students suitable for smart construction is to enhance their engineering innovation abilities and knowledge and develop a greater understanding of the opportunities associated with adopting new technologies [39, 46, 47].
- International ability: internationalization is the process of integrating an international or intercultural dimension into the teaching, research, and service functions of a higher education institution [48, 49]. At present, there is no common definition of internationalized students in the academic field. Thus, this study summarized the international ability of students as intercultural communication ability, cooperation ability, international thinking, and vision, as well as global awareness of international competencies [35, 50, 51].
- Engineering thinking ability: engineering thinking is a form of “invisible” consciousness activity, which is a kind of nonlogical and comprehensive way of thinking adopted by engineering students based on the elements of engineering philosophy and engineering knowledge for the purpose of planning engineering entities [52–54]. The engineering thinking abilities of engineering students suitable for smart construction include interrogative and systematic thinking, critical thinking, and the ability to solve complex engineering problems [32, 39].
- Lifelong learning ability: lifelong learning ability is the prerequisite to ensuring the continuous development and improvement of smart construction engineering students. Engineering students must have the awareness and ability of lifelong learning in order to adapt to the development of industrial technology and meet the requirements of modern production systems [55]. Lifelong learning refers to having an awareness of independent and lifelong learning and the ability to continuously learn and adapt to development. The specific connotations are (1) the ability to recognize the necessity of independent and lifelong learning in the context of social development and (2) the ability to learn independently, including the ability to understand technical problems and summarize and ask questions [39].
- Interdisciplinary ability: with the emergence of new industrial clusters, an education to develop an interdisciplinary ability for engineering students to meet the needs of economic development and smart construction is an emerging trend in the construction industry [56, 57]. It is the ability to integrate resources across disciplines while breaking through the limitations of individual knowledge boundaries [38, 58]. This includes the ability to think across disciplines and also integrate knowledge across disciplines [59, 60].
- Knowledge of engineering: engineering students suitable for smart construction need to master the basic theoretical knowledge necessary for the main engineering disciplinary area in a systematic manner [61]. The engineering knowledge necessary for smart construction students includes mathematics, natural science, engineering fundamentals, and expertise, which are useful for solving complex engineering problems [39, 62]. Smart construction education also needs to include the basic theory and knowledge of related disciplines, such as materials science, mechanical engineering, and digital skills and knowledge, so as to achieve the integration of information technology and engineering knowledge [1, 63].
- Engineering skill: professional skills of engineering students suitable for smart construction include both hard and soft skills. Hard skills refer to generic skills in specified engineering majors, such as design and development, application of modern tools, project management and financial analysis, research and study, and engineering analysis [31, 32, 64], while soft skills include exploring and management skills as well as communication skills [65–67].

### 2.2. Linguistic Landscape

Landry and Bourhis [68] were the first to introduce and use the concept of “linguistic landscape” and defined it as “the language of public road signs, advertising billboards, street names, place names, commercial shop signs, and public signs on government buildings combines to form the linguistic landscape of a given territory, region, or urban agglomeration.” The linguistic landscape has two main types of functions, namely, informative and symbolic. The informative function of language signs indicates the borders of the territory inhabited by a linguistic group and also the availability of a specific language to communicate in that territory. On the contrary, the symbolic function refers to the perception that members of a language group have of the value and status of their languages as compared to other languages [68].

Since the concept of linguistic landscapes was proposed, many researchers have conducted studies on the subject, which reached a climax in 2006. In 2015, John Benjamins, a well-known Dutch publishing group, launched “Landscapes: An International Journal,” marking the maturation of the study of linguistic landscapes [69, 70].

The traditional topics on linguistic landscape include the spread of English, the phenomenon of multilingualism, the gap between language policy and concrete implementation, and the vitality of minority languages that continue to attract academia's attention [71], while new research areas have also emerged, including the linguistic landscape in virtual space and multimodal data-semiotic landscapes, such as [72–74].

Digital linguistic landscape is a new topic, and there is little relevant research on the matter, and this includes the virtual linguistic landscape. Virtual linguistic landscape or cyber linguistic landscape can be viewed as innovative subareas of the digital linguistic landscape. The development of virtual linguistic landscape research is still slow and mostly concentrates on exploring the presentation mode and linguistic forms of virtual linguistic landscape.

### 2.3. Digital Linguistic Landscape

With the development of society and technology, eye tracking, virtual reality (VR), and other technologies have enabled people to extend their understanding of social space from “real space” to “virtual space” [75, 76]. The linguistic landscape with a multimodal combination of sound, picture, image, and color has become an emerging form of expression [77, 78]. In addition, with the growth of multilingual capabilities in digital communication, multilingual options in virtual spaces have become more popular, and linguistic landscapes can be defined not only in physical spaces but also in virtual spaces, such as electronic spaces, popular culture, and the Internet [79].

Digital linguistic landscape refers to the linguistic landscape in digital space. In a microsense, it is a controlled linguistic landscape space formed by combining traditional public road signs, billboards, and store signs with text, images, images, and 2D codes using various digital technologies. In a macrosense, it is the process, method, and technology of collecting, monitoring, analyzing, simulating, creating, and reproducing linguistic landscape information with the help of computer technology, multimedia technology, Internet technology, AI, VR, simulation and sensing technology, and other digital technologies. From a technical perspective, digital linguistic landscape is different from the traditional signage to express the linguistic landscape.

Digital linguistic landscape is an information platform for displaying linguistic landscapes through digital technology. Further, it is a tool for sharing language information. The information provided by digital linguistic landscapes is rich, diversified, and internationalized, and its media are diverse, such as digital websites, digital signage, digital media, and other media [80, 81]. Among them, digital signage refers to the multimedia professional audio-visual system that releases business, financial, and entertainment information through large-screen terminal displays in large shopping malls, supermarkets, hotel lobbies, restaurants, theaters, and other public places [82, 83].

Digital linguistic landscapes not only present linguistic information from around the world and are an important gateway to knowledge, information, and skills but also are tools for people to understand the cultures of various countries and enrich their understanding of the world [73, 84]. Digital linguistic landscape is an extension of the physical linguistic landscape, which also has informative and symbolic functions. The informative function of the digital linguistic landscape includes information delivering function, information indicating function, and information mediation function. The information delivering function is manifested in the form of linguistic information at an intuitive level [85]; the information indicating function is manifested in the form of multimodal linguistic landscapes that serve as an aid to comprehension or emphasis [86]; and the information mediation function is manifested in the form of short, guiding messages that point to a broader pool of relevant information resources [74]. The symbolic function of digital linguistic landscape refers to its ability to reflect market trends and current social conditions [87, 88], as the information of digital linguistic landscape is shared, convenient, and expandable to facilitate the wide dissemination of social phenomena [89], and its information most directly reflects often the most cutting-edge social hotspots and trends. Besides, the information dissemination medium relies on the latest digital technology. Based on these features, digital linguistic landscape is a carrier that reflects current situations and market trends.

## 3. A Priori Model and Hypotheses

### 3.1. Theoretical Supporting Model: ASK

The supporting model “ASK” (also known as KSA) was applied to investigate the mechanism of the digital language landscape on the education of engineering students in smart construction. ASK, a benchmark of the American Council on Education, primarily aimed to assess the capabilities of a prospective job applicant [90]. In federal personnel guidance, KSAs are defined as the factors that identify higher qualified candidates from a group of individuals with varying levels of skills and knowledge [91, 92]. At present, the ASK model is mainly applied to the education of enterprise personnel and management, and it has also been explored in higher education and training of medical human resources. For example, at the level of personnel training, Stahl and Luczak [93] investigated personnel planning in concurrent engineering with KSA. From the perspective of higher education, Hu et al. [94] investigated the impact of the KSA competency enhancement framework on nonengineering students' competencies.

**Figure 1:**
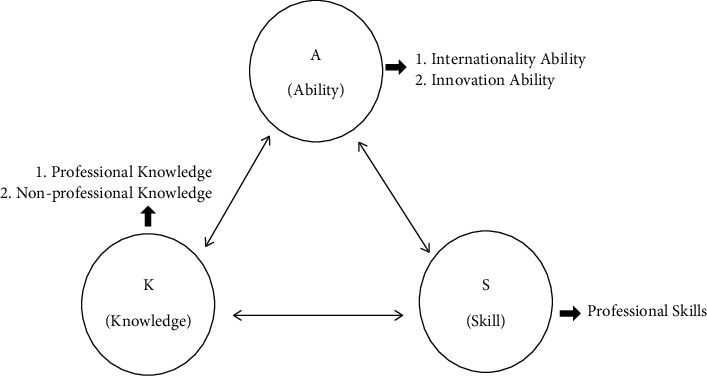
ASK education model for smart construction engineering.

In the ability dimension of the model, this study examined innovation ability and international ability. The ASK model constructed in this study is shown in Figure 1.

### 3.2. Hypotheses

Based on the ASK education model for smart construction engineering, the hypotheses of the mechanism of digital linguistic landscape for smart construction education are proposed.

#### 3.2.1. Digital Linguistic Landscape and the Smart Construction Ability of Engineering Students

With the advancement of China's new era of industrialization, an important mission of higher education institutions focused on engineering education is to cultivate innovative engineering students and provide support for the development of industrialization so as to meet the demand for continuous innovation. Innovation ability refers to the ability of people to discover new problems, propose new ideas, and new ways to solve problems through innovative thinking activities and produce new products, new technologies, or new methods through innovative and practical activities on the basis of rich knowledge and broad horizon [95]. Innovation ability is mainly a comprehensive ability formed by the interaction of such elements as knowledge horizon, innovation awareness, innovation thinking, and innovation skills [46]. Digital linguistic landscapes carry information that is diverse in presentation and rich in content, which helps to enrich knowledge and broaden horizons. It also has the potential to influence the acquisition and development of abilities at the consciousness level through the expansion of information and the diversification of information channels. Thus, the following research hypothesis was proposed regarding the digital linguistic landscape's role in the development of innovation ability for engineering students suitable for smart construction:

  H1: digital linguistic landscapes have a significant positive impact on the innovation ability of engineering students suitable for smart construction.

This study classifies intercultural communication ability, global awareness, and global vision as international abilities. Global vision requires students to be able to think and deal with problems from the perspective of global industrial development and seek opportunities for international development in a complex and changing international environment [96, 97]. Global awareness is a habit of mind that focuses on understanding and grasping the trends of the times [98]. Intercultural communication skills of smart engineering students cannot be developed without knowledge input and the practices of global engineering programs and immersive research experience abroad [99]. From the informative dimension of the digital linguistic landscape, it covers a wide range of information on humanities and history at home and abroad, as well as a wide range of languages, providing a global range of resources and a wide range of channels for inputting knowledge. From the symbolic dimension, the digital linguistic landscape provides a convenient medium to obtain information, a channel to understand the international market situation, and a facilitator to develop international thinking habits. Thus, the following research hypothesis was proposed regarding the digital linguistic landscape's role in the development of international ability of engineering students suitable for smart construction:

  H2: digital linguistic landscapes have a significant positive impact on the international ability of engineering students suitable for smart construction.

#### 3.2.2. Digital Language Landscape and Knowledge Acquisition by Engineering Students Suitable for Smart Construction

The education of engineering students in developed countries focuses on improving the professional knowledge and comprehensive quality of engineers and involves a wide range of courses covering multidisciplinary knowledge [100, 101]. The education mode of engineering students in the United States emphasizes liberal education in humanities, mathematics, and science. This involves a basic education in many disciplines with the goal of educating general engineering students, focusing on the integration of arts and science, enabling an adequate coverage of science and engineering, and supporting interdisciplinary [102, 103], whereas the education mode of engineering students in German universities mainly emphasizes the combination of basic education in humanities and social sciences and professional knowledge education and focuses on practical engineering education, with the goal of educating senior engineering students with a broad knowledge base [104–106].

A research report of the Chinese Academy of Engineering identified that engineering and technology students need to have more professional science and technology awareness and more solid engineering expertise as well as humanities and social knowledge [38]. Indeed, it can be seen that engineering students suitable for smart construction need not only to have the theoretical knowledge of their professions but also to learn the basic knowledge of multidisciplinary, humanities, and social sciences. Since the informative dimension of the digital linguistic landscape provides rich knowledge and information and basic knowledge of humanities and social sciences, as well as interdisciplinary knowledge, it is therefore able to play a key role in expanding the knowledge horizon of engineering students. In terms of the symbolic dimension, the digital linguistic landscape has the characteristics of information sharing, convenience, and expansion, which has the potential to impact the knowledge learning ability of students. Thus, the following research hypothesis was proposed regarding the digital linguistic landscape's role in the education of knowledge acquisition of engineering students suitable for smart construction:

  H3: digital linguistic landscapes have a significant positive impact on the knowledge acquisition of engineering students suitable for smart construction.

#### 3.2.3. Relationship between Digital Linguistic Landscape and Skills Training of Engineering Students

The overall scale and development level of China's construction industry continues to grow, but the construction industry still suffers from low skill quality of construction industry workers, imperfect skill education system, shortage of highly skilled personnel, and other problems, which seriously restricts the transformation of the construction industry. The provision of professional and systematic theoretical knowledge is an effective support to cultivate the skills of construction students [107, 108]. Indeed, China's Ministry of Education [39] proposed that, on the basis of the necessary basic theoretical knowledge and expertise, students should master the basic ability and basic skills to engage in the practical work in their professional fields. Therefore, in order to improve the skills mastery of smart construction engineering students, it is necessary to strengthen their professional theoretical knowledge base. As a platform for information provision and a medium for knowledge sharing, the digital language landscape plays a supporting role in the acquisition of skills and theories. Hence, the following research hypothesis was proposed regarding the digital linguistic landscape's role in the skill training of engineering students suitable for smart construction:

  H4: digital linguistic landscapes have a significant positive impact on the skills training of engineering students suitable for smart construction.

#### 3.2.4. The Dimensions of Digital Language Landscape

Digital linguistic landscapes have informative and symbolic functions. The knowledge dimension in the ASK model derives from the informative function of the digital linguistic landscape, which provides the source and support for the K in the ASK model. The symbolic function of the digital linguistic landscape can provide directions for the development of abilities and skills by reflecting current conditions and market trends. In view of this, the following research hypothesis was proposed on the role dimensions of digital linguistic landscape:

  H5: informative dimension and symbolic dimension are two main aspects of the DLL in the education of engineering students suitable for smart construction.

**Figure 2:**
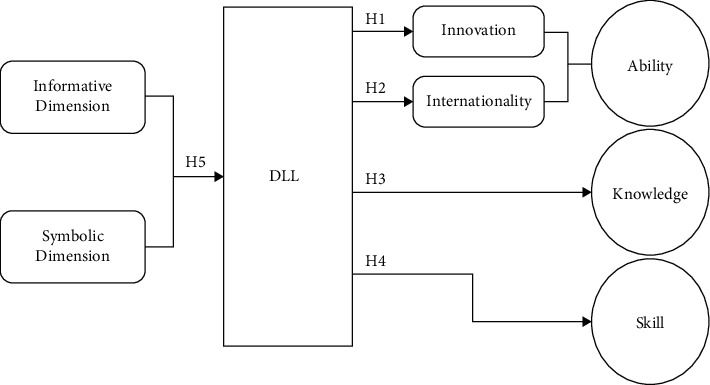
DLL-ASK model.

Based on the above hypotheses, the DLL-ASK model of the mechanism of digital language landscape on smart construction student educating was constructed, as shown in Figure 2.

## 4. Methods

### 4.1. Survey Design, Sample, and Procedure

A questionnaire was designed to survey the relations between DLL and engineering education. The questionnaire consists of two main parts, with four questions focusing on the background of participants and thirty-six statements to measure international ability, innovation ability, knowledge, skills, and informative and symbolic dimension of DLL (see Appendix). For the items in the second part, a 5-point Likert scale was used, with 1 indicating “strongly disagree” and 5 indicating “strongly agree.”

The survey was designed and administered to collect data from September 28, 2021, to October 15, 2021, and was conducted in the form of online distribution on Sojump, a questionnaire platform. The survey was randomly sent to engineering college students, engineering professional teachers, and practitioners. In order to ensure the authenticity and objectivity of the survey data, the questionnaire was answered anonymously, and the purpose and the confidentiality of the study were informed in advance to reduce the privacy concerns of the survey respondents. A total of 193 responses were received.

As can be seen from Table 2, among the participants in the questionnaire survey, 1.05% of them are doctoral students and above, 10.47% are master's students, and 98.43% are bachelor's students and above, which reflects that the overall education level of the respondents in this survey is high. The statistics on the level of intercultural communication ability show that those who said they are not fluent in English account for the most (64.77%), while those who are fluent account for only 30.57%, and 4.66% said they could not communicate at all. This highlights that the overall intercultural communication level of college engineering students is low.

### 4.2. Data Analysis

#### 4.2.1. Treatment of Data

In order to ensure the quality of data collected before commencing data analysis, all the completed questionnaires (*N* = 193) were checked against systematic response patterns and more than 5% missing items, as suggested by [109–111]. All questionnaires were completely answered. In view of the small proportion of engineering teachers and practitioners, their relevant data and questions (2nd question, years of working) were excluded, so 2 out of 193 completed questionnaires were dropped from the data set. To code responses for data analysis, the researchers identified each item as being favorable or unfavorable toward its factor to be measured, as suggested by Seo [109]. Items that score lower than 3 represent unfavorable; otherwise, they represent favorable.

#### 4.2.2. Statistical Analysis

Data were analyzed with structural equation modeling (SEM) procedures, and MPLUS software was used to construct the SEM analysis of the DLL-ASK mechanism. This study used different types of indexes of overall fit for evaluating SEM models, including *x*^2^/degrees of freedom ratio (*x*^2^/df), Comparative Fit Index (CFI), Tucker-Lewis Index (TLI), and the root mean square error approximation (RMSEA).

*(1) Absolute Fit Indexes*. Absolute fit indexes typically evaluate “badness of fit.” *x*^2^/df and root mean square error of approximation (RMSEA) are two commonly used absolute fit indexes.

The model is regarded as acceptable, if the value of *x*^2^/degrees of freedom ratio is less than two. MacCallum et al. [112] suggested that a value of 0.01, 0.05, and 0.08 of RMSEA indicates excellent, good, and mediocre fit, respectively.

*(2) Incremental Fit Indexes*. Incremental fit indexes typically evaluate “goodness of fit,” as larger values indicate a better fit between hypothesized model and data. Commonly used incremental fit indexes include Bentler and Bonett's Normed Fit Index (NFI), Comparative Fit Index (CFI), Tucker-Lewis Index (TLI), and the Incremental Index of Fit (IFI), among which CFI and DFI were used in this study. A CFI value > 0.95 (ranging from 0.00 to 1.00) was considered representative of a well-fitting model. TLI value close to 0.95 represents indicative of a good fit [113].

## 5. Results

### 5.1. Descriptive Statistics

Correlations among all the dimensions are reported in Table 3, showing that all variables were significantly correlated (*p* < 0.05), and none of the correlation values exceeds the threshold value of 0.9, which suggests that the multicollinearity problem does not exist between the items [114].

### 5.2. Exploratory Factor Analysis

KMO and Bartlett's sphericity tests were conducted using SPSS software, and the results are shown in Table 4. The KMO value of the questionnaire sample was 0.864, and Bartlett's test value was less than 0.001, indicating that the sample is suitable for exploratory factor analysis (EFA) and the data information could be extracted effectively.

This study used SPSS24.0 software for the EFA to identify the dimensionality of the survey. After reducing the dimensionality of 36 factors, a total of 6 common factors were extracted, as shown in Table 5, and the cumulative variance explained by the extracted 6 factors was 70.614%, which proved that the extracted 6 factors could explain the original data of the questionnaire well.

Rotated component matrix result is shown in Table 6, and it can be seen that the classification of each question item corresponds exactly to the dimensional settings, indicating that the settings of each question item under the six dimensions are reasonable.

### 5.3. Testing the Measurement Model

The reliability of variables can be tested by Cronbach's alpha coefficient if *α* > 0.6, indicating that the dimension is valid, and the larger it is, the better its reliability is. The results of the reliability test using SPSS software are shown in Table 7. It can be seen that the *α* values of variables are all greater than 0.7, and the CITC values of all measurement items are greater than 0.3, which shows that the data of each variable meet the requirements of reliability.

Then, the validity of factors was tested by confirmatory factor analysis (CFA). The CFA results are shown in Table 8. The results showed that the KMO values of these research variables are higher than 0.7, and Bartlett's test value is less than 0.001, representing statistically significant. Meanwhile, the factor loadings of items are all larger than 0.5, and the cumulative variance explained is above 50%. It represents each measurement item that belongs to the corresponding variable, and the dimension has good discriminant validity and convergent validity and meets the requirements needed for the study, so it can be tested by SEM.

### 5.4. Structural Model

**Figure 3:**
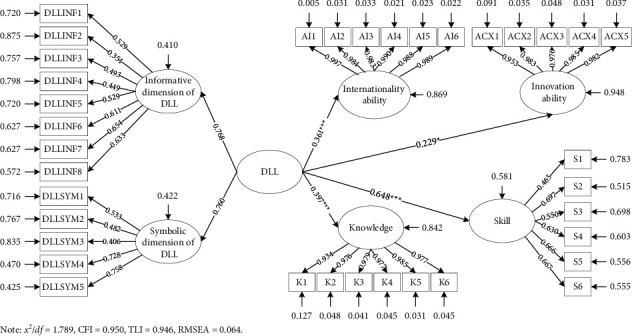
SEM results. *Notes*. *x*^2^/df = 1.789, CFI = 0.950, TLI = 0.946, and RMSEA = 0.064. ^*∗*^*p* < 0.05; ^*∗∗∗*^*p* < 0.001.

The results of SEM analysis of the DLL-ASK mechanism are shown in Figure 3, and the specific coefficient results are shown in Table 9. The results of the SEM fit index are within the acceptable range, as *x*^2^/df is 1.789 < 3, CFI is 0.950 > 0.90, TLI is 0.946 > 0.90, and RMSEA is 0.064 < 0.80. It can be seen that the DLL-ASK model constructed in this paper fits well.

According to the results of the SEM in Figure 3 and Table 9, it was found that the digital linguistic landscape contains an informative dimension and a symbolic dimension with factor loadings of 0.768 and 0.760, respectively. At the same time, the digital linguistic landscape variable consisting of these two dimensions has a positive effect on international ability, with a path coefficient of 0.361 (*p* < 0.001), and also a positive effect on innovation ability, with a path coefficient of 0.229 (*p* < 0.05), assuming that path H1 and H2 are established. Among them, the digital linguistic landscape has more influence on the formation of international ability. In addition, there is a significant positive effect of digital linguistic landscape on knowledge, with a path coefficient of 0.397 (*p* < 0.001), and the same to skill, with a path coefficient of 0648 (*p* < 0.001), proving that the hypotheses paths H3 and H4 are tenable. Considering the effect of digital linguistic landscape on ability, this result shows that digital linguistic landscape has the most significant effect on skills training of engineering students suitable for smart construction.

The hypothesis H5 that the digital linguistic landscape contains both informative and symbolic dimensions holds true, and the digital linguistic landscape combines together the two dimensions to educate engineering students suitable for smart construction. Features 6 (providing rich linguistic information), 7 (providing domestic and foreign human history), and 8 (providing interdisciplinary knowledge) of the informative dimension play the most significant role, and features 4 (reflecting foreign market trends) and 5 (reflecting domestic market trends) of the symbolic dimension play the most obvious role.

## 6. Discussion

This study aims to test the mechanism of the digital linguistic landscape in engineering education for smart construction. The results underscore the importance of digital linguistic landscape in educating engineering students in smart construction. DLL plays a significant role in educating the international ability and innovation ability of engineering students suitable for smart construction.

The findings have significant implications for the specification of the education objectives in regard to the ASK dimensions. The results identify the acting path of DLL on ability, knowledge, and skill education. To be specific, the path to enhance the international ability of engineering students for smart construction is mainly to improve the international competitiveness, cross-cultural communication ability, and broad international horizon. In addition, the path to enhance the innovation ability of the students is mainly to improve the innovation awareness, innovation thinking, and innovation skills, whereas the path to educate the underpinning knowledge of the students is mainly to enrich students' knowledge reserve, broaden their knowledge horizon, and enhance knowledge learning motivation. Furthermore, the improvement of the personal skill level of engineering students is of primary importance in skills training.

The innovation ability is an indispensable ability for engineering students suitable for smart construction [12, 95]. Digital linguistic landscape influences innovative thinking through feature 1 of the informative dimension (extensive information access) and features 2 and 3 of the symbolic dimension (reflecting market trends), innovative awareness through feature 5 of the informative dimension (diverse forms of information presentation), and innovative skills through feature 1 (helping to understand digital technology) of the symbolic dimension. Digital linguistic landscape influences innovative skills through integrating the informative dimension and the symbolic dimension to cultivate the innovation ability of engineering students suitable for smart construction.

Digital linguistic landscape enhances cross-cultural communication ability through feature 6 (providing rich linguistic information) in the informative dimension and broadens international vision through feature 8 (rich interdisciplinary knowledge). Also, DLL improves international competitiveness through features 4 and 5 (reflecting domestic and international current conditions) in the symbolic dimension and integrates the informative dimension and symbolic dimension work together to cultivate the international ability of engineering students suitable for smart construction. Cultivation of the international ability of engineering students suitable for smart construction enables the students to become the leaders of the construction industry of the future [15].

Digital linguistic landscape educates the knowledge dimension of smart construction engineering students by enriching the professional and nonprofessional knowledge reserve of students through feature 4 (providing diversified foreign language information) and feature 6 (providing rich languages) of the informative dimension of digital linguistic landscape and broadens the knowledge horizon through feature 8 (rich interdisciplinary knowledge) of the informative dimension and features 2–5 of the symbolic dimension (reflecting market trends and current conditions). To broaden the knowledge horizons and enhance knowledge learning motivation through informative dimension feature 2 (easy access to information) and feature 5 (multimodal information presentation form) allow integration of two dimensions to cultivate the knowledge dimension of smart construction engineering students.

The specific path of the digital linguistic landscape for the skill education of engineering students suitable for smart construction is to train the skill of smart construction students through features 2 (easy access to information), feature 4 (providing multiple foreign language information), and feature 6 (providing rich language information) of the informative dimension of digital linguistic landscape.

Educating the ability of engineering students in smart construction is the driver and support for the development of the smart construction industry [115]. Knowledge has been identified as a key driving force for innovation [95]. Thus, the development of the knowledge dimension also indirectly acts on the improvement of the innovation ability. By improving the skill level, smart construction students can improve their skill level and thereby be more able to adapt to society and meet its changing needs more quickly [99]. Therefore, it is necessary to strengthen the application of DLL in engineering education.

## 7. Conclusion

This research investigates the influence factors on the education of engineering students suitable for smart construction. The study divided the qualities of engineering students suitable for smart construction into the three dimensions of ability, knowledge, and skill based on the ASK model, and a DLL-ASK hypothesis model was subsequently constructed. Based on this approach, the DLL-ASK model was surveyed and tested using a questionnaire and SEM.

The results of the study identify the following: (1) The informative dimension and symbolic dimension are two main aspects of the DLL in engineering education for smart construction. (2) DLL has a significant positive impact on the ability, knowledge, and skills of engineering students suitable for smart construction. (3) DLL educates the ability, knowledge, and skills of engineering students for smart construction by transmitting rich language information and multilingual information as well as reflecting market trends at home and abroad. The research results are consistent with the DLL-ASK hypothesis model, and therefore, the DLL-ASK model is supported.

Based on the above results, the following suggestions are made on how to educate engineering students for smart construction from the informative dimension and symbolic dimension of the digital linguistic landscape:

1. Increase the amount of digital linguistic landscapes in engineering education. It is highly important to enhance the use and edification of digital linguistic landscape in the real engineering student training environment or digital space learning environment. Through strengthening the edification and influence of digital linguistic landscape in smart construction training, it is more likely to gradually improve the ability and skills of students and broaden their knowledge horizon as well. Specific measures include increasing the amount of digital signage on campus, using digital media to support the education process, and establishing a digital linguistic landscape network platform for information sharing.
2. Pay more attention to the content of digital language landscape information. Digital language information is most directly accessed and absorbed by students, so this feature can be harnessed to present the education contents to smart construction engineering students through the carrier of the digital language landscape. For example, for the cross-cultural communication ability of engineering students, the skills to enhance abilities or access to resources can be presented through multimodal presentation such as voice, image, and QR code.
3. Make full use of digital language landscape technology guidance role. As the embodiment of digital technology, digital language landscape has a significant impact on the technical development of engineering students for smart construction. By learning the application of digital technology, smart construction students can better and faster integrate the trend of social development and meet the changing needs of the construction industry. Therefore, it is necessary to periodically update the digital language landscape medium and use the latest technology-supported digital signage so as to connect digital technology with smart construction technology.

The limitation of this study is that although the participants appear to be representative of engineering students in China's higher education, there is nevertheless a lack of involvement of teachers and practitioners. Moreover, the participants were limited to Chinese engineering students, and there was a lack of involvement of international students. Therefore, future research should adopt a larger sample size with a wider range of participants to further examine the external validity of the study.

## Figures and Tables

**Table 1:** Core qualities of engineering students.

	A	B	C	D	E	F	G
FEANI [30]	✓		✓			✓	✓
Sydney Accord [31]	✓		✓	✓	✓	✓	
Washington Accord [32]	✓		✓		✓	✓	✓
IEC [33]	✓		✓	✓	✓	✓	✓
Cutler et al. [34]		✓	✓		✓	✓	✓
Klein-Gardner et al. [35]			✓		✓	✓	
Siller et al. [36]		✓	✓		✓	✓	✓
Stieff et al. [37]		✓	✓				
Hu and Li [38]	✓	✓				✓	✓
CEEAA [39]	✓		✓	✓	✓	✓	✓
Proposed index in this research		✓	✓		✓	✓	✓

*Notes*. A, lifelong learning ability; B, innovation ability; C, international ability; D, engineering thinking ability; E, interdisciplinary ability; F, Knowledge; G, Skill.

**Table 2:** Sample information table.

Items	Options	%
Degree	Associate/bachelor	86.91
	Master	10.47
	Doctor and above	1.05

English level	Else	1.57
	Very fluent	1.55
	Fluent	29.02
	Not fluent	64.77
	Cannot speak at all	4.66

**Table 3:** Correlations among variables.

Dimensions	A-I	A-CX	*K*	*S*	DLL-Info	DLL-Sym
A-I	1					
A-CX	0.264^*∗∗*^	1				
*K*	0.560^*∗∗*^	0.151^*∗*^	1			
*S*	0.225^*∗∗*^	0.203^*∗∗*^	0.255^*∗∗*^	1		
DLL-Info	0.318^*∗∗*^	0.233^*∗∗*^	0.263^*∗∗*^	0.359^*∗∗*^	1	
DLL-Sym	0.222^*∗∗*^	0.187^*∗*^	0.305^*∗∗*^	0.412^*∗∗*^	0.525^*∗∗*^	1

^*∗∗*^Correlation is significant at the 0.01 level. ^*∗*^Correlation is significant at the 0.05 level.

**Table 4:** KMO and Bartlett's test.

KMO and Bartlett's test		
KMO		0.864
Bartlett test of sphericity	Approx. Chi-square	9892.396
	df	1081
	Sig.	0.000

**Table 5:** Common factors.

Total variance explained									
Component	Initial eigenvalues			Extraction sums of squared loadings			Rotation sums of squared loadings		
	Total	% of variance	Cumulative %	Total	% of variance	Cumulative %	Total	% of variance	Cumulative %
1	10.903	30.287	30.287	10.903	30.287	30.287	5.910	16.416	16.416
2	4.603	12.787	43.074	4.603	12.787	43.074	5.776	16.044	32.460
3	3.800	10.556	53.629	3.800	10.556	53.629	4.878	13.549	46.009
4	2.688	7.467	61.097	2.688	7.467	61.097	3.241	9.004	55.013
5	2.010	5.583	66.680	2.010	5.583	66.680	3.071	8.530	63.543
6	1.416	3.934	70.614	1.416	3.934	70.614	2.545	7.071	70.614
7	1.169	3.247	73.861						
8	0.975	2.709	76.570						
9	0.917	2.548	79.118						
10	0.869	2.413	81.531						
11	0.814	2.260	83.791						
12	0.718	1.995	85.786						
13	0.641	1.780	87.566						
14	0.603	1.674	89.240						
15	0.572	1.590	90.830						
16	0.555	1.542	92.372						
17	0.495	1.374	93.746						
18	0.435	1.209	94.955						
19	0.401	1.113	96.068						
20	0.351	0.975	97.043						
21	0.340	0.944	97.987						
22	0.230	0.638	98.625						
23	0.102	0.283	98.907						
24	0.076	0.210	99.118						
25	0.048	0.133	99.251						
26	0.044	0.123	99.374						
27	0.038	0.105	99.479						
28	0.033	0.092	99.571						
29	0.032	0.089	99.660						
30	0.025	0.070	99.731						
31	0.025	0.069	99.799						
32	0.023	0.063	99.863						
33	0.018	0.050	99.912						
34	0.015	0.042	99.954						
35	0.011	0.031	99.985						
36	0.005	0.015	100.000						

Extraction method: principal component analysis.

**Table 6:** Rotated component matrix.

Items	Components					
	1	2	3	4	5	6
A-I1	0.946					
A-I2	0.928					
A-I3	0.922					
A-I4	0.926					
A-I5	0.934					
A-I6	0.933					
A-CX1			0.956			
A-CX2			0.966			
A-CX3			0.962			
A-CX4			0.971			
A-CX5			0.965			
K1		0.917				
K2		0.924				
K3		0.933				
K4		0.907				
K5		0.923				
K6		0.925				
DLL-Info1				0.542		
DLL-Info2				0.376		
DLL-Info3				0.362		
DLL-Info4				0.543		
DLL-Info5				0.657		
DLL-Info6				0.725		
DLL-Info7				0.656		
DLL-Info8				0.602		
DLL-Sym1						0.486
DLL-Sym2						0.388
DLL-Sym3						0.341
DLL-Sym4						0.761
DLL-Sym5						0.850
S1					0.566	
S2					0.730	
S3					0.602	
S4					0.682	
S5					0.690	
S6					0.606	

Extraction method: principal component analysis.

**Table 7:** CITC and Cronbach's *a* coefficient of variables.

Dimensions	Variables	Items	CITC	*α*
Ability: international ability	A: A-I	A-I1	0.995	0.976
		A-I2	0.983	
		A-I3	0.980	
		A-I4	0.985	
		A-I5	0.987	
		A-I6	0.985	

Innovation ability	A-CX	A-CX1	0.947	0.928
		A-CX2	0.975	
		A-CX3	0.967	
		A-CX4	0.976	
		A-CX5	0.973	

Knowledge	K	K1	0.930	0.972
		K2	0.971	
		K3	0.975	
		K4	0.972	
		K5	0.979	
		K6	0.972	

Skill	S	S1	0.405	0.781
		S2	0.618	
		S3	0.465	
		S4	0.551	
		S5	0.582	
		S6	0.557	

Informative dimension of DLL	DLL-Info	DLL-Info1	0.461	0.763
		DLL-Info2	0.323	
		DLL-Info3	0.396	
		DLL-Info4	0.431	
		DLL-Info5	0.489	
		DLL-Info6	0.525	
		DLL-Info7	0.532	
		DLL-Info8	0.513	

Symbolic dimension of DLL	DLL-Sym	DLL-Sym1	0.450	0.708
		DLL-Sym2	0.382	
		DLL-Sym3	0.345	
		DLL-Sym4	0.539	
		DLL-Sym5	0.614	

**Table 8:** Confirmatory factor analysis results of variables.

Dimensions	Variables	Items	Factor loadings	KMO	*P*	Explained variance ratio (%)
Ability: international ability	A: A-I	A-I1	0.987	0.927	<0.001	98.110
		A-I2	0.991			
		A-I3	0.990			
		A-I4	0.990			
		A-I5	0.988			
		A-I6	0.996			
Innovation ability	A-CX	A-CX1	0.967	0.927	<0.001	96.154
		A-CX2	0.985			
		A-CX3	0.984			
		A-CX4	0.981			
		A-CX5	0.967			
Knowledge	K	K1	0.951	0.946	<0.001	95.320
		K2	0.982			
		K3	0.981			
		K4	0.979			
		K5	0.978			
		K6	0.951			
Skill	S	S1	0.960	0.818	<0.001	74.118
		S2	0.615			
		S3	0.887			
		S4	0.888			
		S5	0.655			
		S6	0.692			
Informative dimension of DLL	DLL-Info	DLL-Info1	0.783	0.768	<0.001	81.749
		DLL-Info2	0.920			
		DLL-Info3	0.939			
		DLL-Info4	0.892			
		DLL-Info5	0.783			
		DLL-Info6	0.838			
		DLL-Info7	0.820			
		DLL-Info8	0.670			
Symbolic dimension of DLL	DLL-Sym	DLL-Sym1	0.599	0.713	<0.001	79.622
		DLL-Sym2	0.958			
		DLL-Sym3	0.912			
		DLL-Sym4	0.900			
		DLL-Sym5	0.855			

**Table 9:** SEM coefficiency results.

Paths	Coefficiency	Standard error	*p* value	95% confidence intervals	Hypotheses
DLL⟶international ability	0.361	0.099	^*∗∗∗*^	[0.168; 0.524]	Support
DLL⟶innovation ability	0.229	0.109	^*∗*^	[0.015; 0.443]	Support
DLL⟶knowledge	0.397	0.093	^*∗∗∗*^	[0.214; 0.550]	Support
DLL⟶skill	0.648	0.115	^*∗∗∗*^	[0.423; 0.836]	Support

*Notes*. ^*∗*^*p* < 0.05; ^*∗∗∗*^*p* < 0.001.

**Table 10:** Questionnaire statements.

Items	Specific statements
1	Understanding domestic and international current conditions can help people enhance one's international competitiveness
2	Understanding market trends helps people enhance international competitiveness
3	Access to information on foreign language knowledge can help enhance one's intercultural communication skills
4	Access to rich linguistic information can help enhance one's intercultural communication skills
5	Access to information on humanities and social sciences helps to develop one's international perspective
6	Access to multidisciplinary knowledge helps to broaden one's global vision
7	Understanding domestic and international current situation helps to enhance one's innovation awareness
8	Understanding market trends helps enhance one's innovative thinking
9	Understanding the latest digital technology helps enhance one's innovative skills
10	Diversified access to information helps enhance one's innovative thinking
11	Diversified forms of information presentation can help enhance one's innovative thinking
12	Access to more language information helps to strengthen one's professional knowledge base
13	Access to foreign language knowledge helps enhance one's nonspecialized knowledge base
14	Understanding multidisciplinary knowledge helps broaden one's knowledge horizon
15	Understanding domestic and international market trends helps broaden one's knowledge horizons
16	Easy access to language information helps to increase motivation to learn
17	Access to a wide range of language information helps to increase motivation to learn
18	Knowledge of the latest digital technologies helps to strengthen one's skills
19	Understanding more knowledgeable information helps to strengthen one's skills
20	Understanding domestic and international current conditions helps strengthen one's skills
21	Knowledge of domestic and international market trends helps strengthen one's skills
22	Knowledge of foreign language information helps strengthen one's skills
23	Easy access to information helps strengthen personal skills
24	DLL can increase people's access tunnels to information
25	DLL makes it easier for people to access information
26	DLL allows people to share information resources
27	DLL multimodality increases people's interest in it
28	DLL provides various languages
29	DLL provides a wealth of linguistic information
30	DLL helps people understand the history of people at home and abroad
31	DLL can provide access to multidisciplinary knowledge
32	DLL provides access to the latest digital technologies
33	DLL provides access to domestic current situations
34	DLL provides access to foreign current situations
35	DLL helps people study foreign market trends
36	DLL helps people study domestic market trends

## Data Availability

The data used to support the findings of the study could be obtained from the corresponding author upon request.

## Appendix

### Questionnaire

Linguistic landscape includes physical landscapes formed by language, such as language appearing on public signs such as public street signs, billboards, street names, place names, and store signboards. It also includes language information on virtual media such as digital signage, electronic billboards, and network video advertisements. Digital linguistic landscape is a platform for displaying linguistic landscape information through digital technology, a medium for carrying linguistic information in virtual space, such as QR codes, digital advertisements, and multimedia videos.

The survey is anonymous, and the data of this questionnaire is for academic research use only. Your honest, personal opinions are greatly appreciated and help us a lot. Thanks for your time!

(I) Demographic traits
  (1). Your identity: □Engineering Practitioner □Engineering Teacher □Engineering Learner
  (2). How long have you been working in this industry: ______
  (3). Your major: ______
  (4). Your education: □university or college □postgraduate □doctor □else
(II) Your evaluations of the following roles' degree

Five choices are listed below each question, from 1 indicating “strongly disagree” to 5 indicating “strongly agree” (Table 10).
